# Coronary artery spasm in cardiac arrest survivors

**DOI:** 10.1093/europace/euag020

**Published:** 2026-02-02

**Authors:** Dorte Stavnem, Priya Bhardwaj, Reza Jabbari, Thomas Engstrøm, Lia Evi Bang, Colin Berry, Juan-Carlos Kaski, Jacob Tfelt-Hansen, Bo Gregers Winkel

**Affiliations:** The Department of Cardiology, The Heart Centre, Copenhagen University Hospital, Rigshospitalet, Blegdamsvej 9, 2100 Copenhagen, Denmark; The Department of Cardiology, The Heart Centre, Copenhagen University Hospital, Rigshospitalet, Blegdamsvej 9, 2100 Copenhagen, Denmark; Section of Forensic Genetics, Department of Forensic Medicine, Faculty of Health and Medical Sciences, University of Copenhagen, Copenhagen, Denmark; The Department of Cardiology, The Heart Centre, Copenhagen University Hospital, Rigshospitalet, Blegdamsvej 9, 2100 Copenhagen, Denmark; The Department of Cardiology, The Heart Centre, Copenhagen University Hospital, Rigshospitalet, Blegdamsvej 9, 2100 Copenhagen, Denmark; The Department of Cardiology, The Heart Centre, Copenhagen University Hospital, Rigshospitalet, Blegdamsvej 9, 2100 Copenhagen, Denmark; School of Cardiovascular and Metabolic Health, University of Glasgow and NHS Golden Jubilee National Hospital, Glasgow, UK; Cardiovascular and Genomics Research Institute, City St George's, University of London, London, UK; The Department of Cardiology, The Heart Centre, Copenhagen University Hospital, Rigshospitalet, Blegdamsvej 9, 2100 Copenhagen, Denmark; Section of Forensic Genetics, Department of Forensic Medicine, Faculty of Health and Medical Sciences, University of Copenhagen, Copenhagen, Denmark; The Department of Cardiology, The Heart Centre, Copenhagen University Hospital, Rigshospitalet, Blegdamsvej 9, 2100 Copenhagen, Denmark

**Keywords:** Coronary artery spasm, Vasospastic angina, Sudden cardiac arrest, Provocative testing, Pharmacological therapy, Implantable cardioverter-defibrillator

## Abstract

Coronary artery spasm can be life-threatening. Clinically significant complications include myocardial infarction, ventricular arrhythmias, and sudden cardiac arrest. Although challenging to diagnose, new international guidelines have been published to guide the diagnosis of coronary artery spasm when this is the suspected cause of cardiac arrest. The aim of this review is to consider existing knowledge for the diagnosis and management of coronary artery spasm in survivors of sudden cardiac arrest.

Twenty-seven original research articles (written in English) involving a total of 1541 survivors of sudden cardiac arrest associated with coronary artery spasm form the basis of this review. Most cohorts included >75% male participants with a mean age range of 45–63 years. A positive family history or coronary risk factors of coronary artery disease are not commonly found, albeit many survivors are smokers (ranged 17–100% across cohorts). Provocative testing for coronary spasm was reported in 25 of the evaluated papers, but the indications for testing were inconsistently specified. A high recurrence rate (up to 45%) of life-threatening ventricular arrhythmias was reported, and implantable cardioverter-defibrillator placement varied markedly.

In conclusion, diagnosing coronary artery spasm as a cause of sudden cardiac arrest is challenging. The pathophysiological understanding is limited. Knowledge gaps include the incidence and prevalence, as well as the usefulness of provocative testing in survivors. More data are needed regarding patient risk stratification, indications for implantable cardioverter-defibrillator insertion, and optimal pharmacological therapy.

## Graphical Abstract

**Graphical Abstract:**
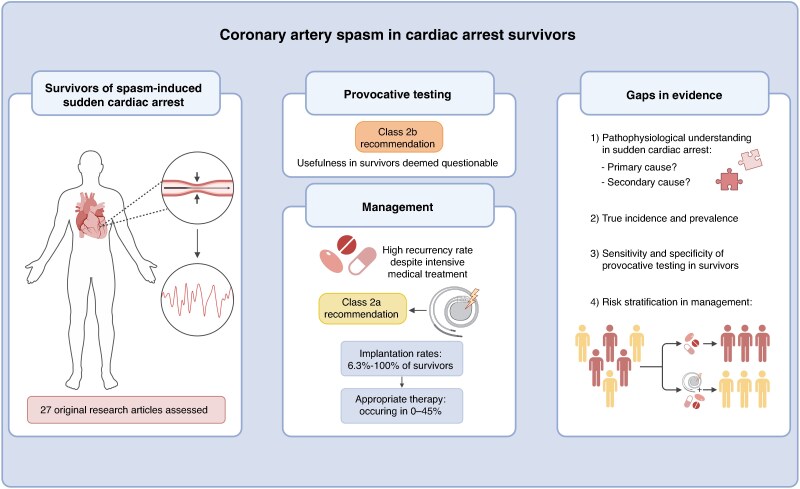

## Introduction

Coronary artery spasm is a vasomotor disorder with diverse clinical presentations. As the pathophysiologic basis of vasospastic angina, it constitutes one endotype of chronic epicardial coronary syndrome,^1,2^ and can equally cause acute coronary syndrome (with and without epicardial stenoses).^3^ The disorder was first described by Prinzmetal *et al*. in 1959 as ‘variant angina’,^4^ yet the pathogenesis is complex and remains incompletely understood.^5^ Evidence suggests that it is mainly due to a hyperactivity of vascular smooth muscle cells possibly involving enhanced Rho-kinase activity and other mechanisms.^6,7^ Endothelial dysfunction and chronic low-grade inflammation may be contributing factors.^1,2,6,7^ Spasm, which can occur in the epicardial arteries or the microvessels, is usually a transient event but can on occasions lead to severe and prolonged myocardial ischaemia.^2,6,8^ Patients with vasospastic angina do not generally have exertional angina. Typically, they present with angina at rest, often between night-time and early morning following a circadian pattern, but the clinical presentation demonstrates great variability. Chest pain can be precipitated by emotional stress, hyperventilation, magnesium deficiency, exposure to cold temperatures, cocaine use, and pharmacological agents, such as ergot alkaloids, triptans, and serotonin-reuptake inhibitors.^1,6,9^ Coronary spasm usually resolves rapidly with the administration of sublingual nitrates.^10^ Diagnosing vasospastic angina is often challenging with diagnostic delays exceeding 6 months in approximately one-third of cases.^11^ This timeline raises concern since coronary artery spasm carries a significant risk of serious adverse cardiovascular events, including acute myocardial infarction, life-threatening ventricular arrhythmias (VAs), and sudden cardiac arrest (SCA).^1,2,6,7,9,12,13^ The aim of this literature review is to present and discuss existing knowledge regarding diagnostic strategies and management in survivors of SCA induced by coronary artery spasm.

## Method

A literature review identified 27 original research articles published in the English medical literature from 1982 to 2025, which assessed survivors of SCA caused by coronary artery spasm. The used search terms were ‘Coronary artery spasm’ OR ‘Vasospastic angina’ OR ‘Coronary vasospasm’ AND ‘Cardiac arrest’ OR ‘Cardiac death’. *Table 1* provides details for each of the publications included in the present review.^14–40^

**Table 1:** Publications on cardiac arrest and coronary artery spasm from 1982 to 2025

Authors	Publication year	Country of origin	Aims of study	Number of studied patients with resuscitated SCA	Number of survivors diagnosed with CAS	Data collection period	Mean follow-up (months)
Miller *et al*.^14^	1982	Canada	Clinical characteristics	13	13	1975–1981	26.2
Fellows *et al*.^15^	1987	USA	Describe association between CAS and SCA	260	6	−	19.6
Myerburg *et al*.^16^	1992	USA	Describe association between CAS and SCA	356, only 13 with unexplained SCA	5	1980–1991	36
Igarashi *et al*.^17^	1992	Japan	Prevalence and patient characteristics	31; only 14 without underlying heart disease	9	1986–1991	31.4
Chevalier *et al*.^18^	1998	France	Patient characteristics and disease evolution	7	7	—	58
Parchure *et al*.^19^	2001	United Kingdom	QT-dispersion in CAS-related CA and syncope	5	5	1992–1998	—
Meune *et al*.^20^	2003	France	Clinical characteristics	300	10	1994–2000	58
Takagi *et al*.^21^	2009	Japan	Prevalence performing ‘dual induction tests’	12	10	2004–2008	19
Takagi *et al*.^22^	2011	Japan	Clinical characteristics and long-term prognosis	365	35	2003–2008	32
Matsue *et al*.^23^	2012	Japan	Clinical significance of ICD treatment	23	23	1999–2011	24.2
Kobayashi *et al*.^24^	2013	Japan	Prevalence and patient characteristics	121	12	2006–2011	—
Togashi *et al*.^25^	2013	Japan	Symptoms and clinical outcomes	18	18	1996–2008	—
Lee *et al*.^26^	2014	South Korea	Predictors of recurrent SCA events	68	68	—	46.8
Yamashina *et al*.^27^	2014	Japan	Clinical features and long-term outcomes	18	18	1992–2012	67
Komatsu *et al*.^28^	2016	Japan	Long-term prognosis and ICD treatment based on ‘dual induction tests’	47 without structural heart disease	31	2004–2014	38
Ahn *et al*.^29^	2016	South Korea	Long-term risk	188	188	1996–2014	90
Rodríguez-Mañero *et al*.^30^	2017	Europe	Clinical outcomes of therapeutics	49	49	—	59
Tateishi *et al*.^31^	2018	Japan	Incidence, clinical outcomes and ECG-changes	474, only 155 without obvious extracardiac cause	17	2011–2015	—
Vlasta *et al*.^32^	2018	Netherlands	Long-term outcomes	11	11	2002–2015	90
Park *et al*.^33^	2018	Korea	Long-term outcomes	598	598	2007–2015	48
Waldmann *et al*.^34^	2018	France	Diagnostic investigation and management	1557	31	2006–2011	48.7^a^
Sueda *et al*.^35^	2020	Japan	Clinical outcomes and provocative testing	169	98	—	66.4
Sueda *et al*.^36^	2020	Japan	Provocative testing	5	5	—	—
Lee *et al*.^37^	2020	Korea	Patient characteristics and clinical outcomes	413	89	2010–2015	1
Tateishi *et al*.^38^	2021	Japan	ACh-testing and overlapping disorders	20	15	2012–2019	38
Park *et al*.^39^	2022	Korea	Significance and prognosis of CAS in ASCD	863	119	2015–2018	—
Tateishi *et al*.^40^	2022	Japan	Prognostic impact of ICD therapy	280	51	2012– 2019	45.6

^a^Note, data on mean follow-up in the study of Waldmann *et al*.^34^ was found in their original article.^41^

ACh, acetylcholine; ASCD, aborted sudden cardiac death; CA, cardiac arrest; CAS, coronary artery spasm; ECG, electrocardiographic; ICD, implantable cardioverter defibrillator; SCA, sudden cardiac arrest.

### Coronary spasm in survivors of SCA—prevalence

In the 27 papers assessed, 1541 patients were considered to be survivors of SCA triggered by coronary artery spasm. Asian studies comprised 18 of the 27 papers, consistent with reports of a higher prevalence of variant angina and coronary artery spasm-related SCA in Asian patients compared to Western populations,^6,42^with Japanese patients suggested to have a three-fold greater incidence.^43^ However, when standardized diagnostic protocols are applied, the frequency and angiographic characteristics of coronary spasm in Western cohorts may approach those observed in Asian populations.^44^ Ethnicity could explain a possible variance, as could difference in awareness and frequency of diagnostic testing.^1^ The Korean Cardiac Arrest Research Consortium revealed that 13.8% of their cohort of survivors of out-of-hospital cardiac arrest had vasospasm identified as a cardiac cause of SCA.^39^ Two studies from Japan found comparable incidence rates of 11% and 13.1%.^31,37^ Meanwhile, one study from Europe identified 31 coronary artery spasm-related SCA from a total of 1557 SCA-cases, indicating an incidence of 2.0% in this population.^34^ The prevalence of coronary artery spasm-related SCA in other Western countries is not known at present, hence no accurate data on incidence and mortality rates exist. Studies looking at postmortem findings (i.e. coronary artery spasticity characterized by pronounced folding of the internal elastic membrane) may be of help.^24,45^

### When to suspect coronary artery spasm in SCA?—Clinical presentation and triggers

Survivors of spasm-related SCA differ substantially from the typical patients with angina with no obstructive coronary arteries (ANOCA). While ANOCA mostly affects women (50–70%), and typically present with mean age of ∼60 years and established cardiovascular risk factors,^46,47^ survivors demonstrate contrasting characteristics: Survivors are predominantly male with ≥75% male composition reported in the majority of study cohorts.^15–17,20–25,27–31,33–37,39,40^ Patient age ranged from a mean of 45 years to 63 years.^15,16,18–25,27,29–31,33–40^ A positive family history or risk factors of coronary artery disease are not commonly found,^17,18,20–22,25,29,37^ albeit many survivors are smokers (ranged 17.4–100% across cohorts).^18,20–25,27,29–31,36,39–41^ Smoking is recognized as a major risk factor for coronary artery spasm,^48^ especially within the Japanese population and in male patients.^43,49^ None of the studies have fully explored potential genetic risk factors, but a recent publication suggests *EDN1* to be a possible genetic risk locus in patients of European ancestry with diffuse epicardial coronary artery spasm.^50^ Compared with controls, patients with coronary artery spasm showed increased levels of plasma endothelin-1,^50^ a peptide known to be a potent coronary vasoconstrictor.^51^

SCA was the first symptom of coronary artery spasm in 67% survivors in one study.^25^ In other publications, up to 80% of survivors did not recall experiencing chest pain prior to their cardiac arrest.^18,20,24^ However, retrograde amnesia may be an important reason for prodromes of angina to be under-reported in the literature.^17^ In cases where survivors did remember experiencing symptoms, chest pain was the most common complaint.^15,17,23,29^ Nocturnal angina appeared less frequently in survivors, indicating a possible different circadian variance compared to coronary artery spasm-patients with only angina.^25^

Comparable numbers of patients (77–86%) are reported to have had SCA during sleep, at rest or during normal activity of daily life.^17,20,27,32^ Conditions regarding the SCA-event is more favourable comparing to survivors of cardiac arrest related to atherosclerotic coronary artery syndrome: coronary artery spasm-survivors had a higher incidence of prehospital return of spontaneous circulation, and a lesser need for intra-aortic balloon pumps, extracorporeal membrane oxygenation, and vasopressors.^37^ They also demonstrated lesser ST-segment elevation but had a higher need for nitrates.^37^

Eight of the evaluated papers applied the ‘Myerburg criteria’ for silent ischaemia for their patient enrolment i.e. (i) documented ventricular fibrillation or sustained ventricular tachycardia, (ii) absence of a previous history of angina pectoris or acute myocardial infarction, (iii) preserved left ventricular ejection fraction and wall motion, (iv) absence of significant coronary artery stenosis of ≥50%, and (v) absence of identifiable or reversible cause of SCA.^16,18,19,23,25,28,30,40^ However, these criteria are not formally established. The Coronary Vasomotion Disorders International Study Group offer standardized diagnostic criteria for epicardial coronary spasm (as requiring nitrate-responsive angina and transient ischaemic electrocardiographic changes in association with documented spasm with >90% vasoconstriction),^52^ yet their application in the post-sudden cardiac arrest investigation remains unclear.

### When to investigate coronary artery spasm invasively—diagnosis

Coronary artery spasm and acute coronary syndrome triggered by obstructive coronary artery disease present with similar clinical features, including electrocardiographic abnormalities and elevated biomarkers for myocardial necrosis.^24^ Therefore, distinguishing between these two causes of SCA requires an invasive diagnostic procedure.^24^ Conversely, the literature provides limited guidance on when coronary artery spasm should be suspected as the cause of SCA. The 2022 European Society of Cardiology (ESC) guidelines on VAs and sudden cardiac death (SCD) recommend investigating coronary artery spasm in SCA-survivors if there is (i) a strong clinical suspicion and (ii) all other tests are normal (a Class 2b recommendation).^53^ This is supported by a novel consensus paper on coronary artery spasm in out-of-hospital cardiac arrest survivors.^54^ In patients fulfilling the two ESC criteria, provocative testing for coronary artery spasm can be carried out using intracoronary acetylcholine (ACh) or ergonovine.^2^ Both provocative agents have a complicated mechanism of action, which primarily act by directly stimulating smooth muscle cells in arteries with endothelial dysfunction.^32^ A test is considered to be ‘positive’ for spasm in the presence of ≥90% luminal narrowing on angiography, accompanied by both ischaemic electrocardiographic changes and chest pain.^3,52,54^ ACh is preferred due to its short-lasting effect,^3^ compared with ergonovine, which may elicit sustained ventricular tachycardia.^55^ However, provocative tests are considered to be safe.^56,57^ Importantly, high ACh-dosages can induce false-positive test results.^58^ Vasoactive medications such as calcium channel blockers (CCBs), and long-acting nitrates should be discontinued at least 24–48 h before provocative testing to avoid false-negative results.^59,60^

In the publications assessed in the present review, provocative testing was performed with ACh in 10^21,23–25,27,28,31,32,38,40^; with ergonovine in 11,^14–20,26,29,34,37^ and with both in 4^22,30,35,36^ and not specified in 2.^33,39^Provocative tests were not performed consistently or uniformly across studies, and indications for testing were not always clear. The definition of a positive test result varied among studies, i.e. spastic lumen narrowing of at least 50%, 75%, or 95%,^17,20,22,30,31^ but not always specified. Provocative testing was considered redundant by the treating physicians in some studies if (i) spasm occurred spontaneously during coronary angiography or (ii) if anginal symptoms were associated with transient ST-segment elevations in the electrocardiogram of patients with angiographically normal coronary arteries. The time from SCA to provocative testing was rarely reported, but when documented, intervals ranged from a mean of 20 days to 1 month after the index event across the available studies.^20,21,38^ The optimal timing and indication for conducting provocative testing are not clear in the literature or specified in current guidelines.

The diagnostic approaches have changed over time,^59^ which makes it difficult to fully understand the role of coronary artery spasm in SCA, especially as the number of cases reported in the literature is relatively small. For example, Miller *et al*. excluded patients with positive ergonovine tests, and solely included patients with observed coronary artery spasm attacks during hospital stay, who had transient electrocardiographic changes but without signs of myocardial necrosis.^14^ Their data collection began in 1975, only 3 years after ergonovine was used for the first time as a provocative agent in the diagnosis of coronary artery spasm. In 1987, the administration of ergonovine as an intracoronary injection instead of intravenously was proven useful. The following year, ACh was reported as a provocative agent for spasm with high sensitivity (90%) and specificity (99%).

While a few studies have commented on a potential link between spasm site and a more malignant phenotype (particularly regarding multivessel spasm and diffuse right coronary artery spasm,^22,25,26,29^) no consensus has been established. The interpretation of provocative test results thus remains complex. A positive test result does not definitively establish causation between coronary artery spasm and SCA, as noted by The Lancet commission on SCD prevention.^61^ A recent consensus statement even suggests it is ‘uncertain if coronary artery spasm testing can be useful in assessing all individuals presenting with unexplained cardiac arrest after comprehensive testing’.^54^ The reliability of ACh has previously been challenged by research showing a 44.2% false-positive rate in 77 patients with a positive ACh test. This was based on the fact that 34 of the patients did not experience rest angina and therefore suggesting that a negative test result should be anticipated.^62^ This cohort consisted of individuals with angina pectoris and former acute myocardial infarction but not resuscitated cardiac arrest. To the best of our knowledge, both the sensitivity and specificity of the ACh test in survivors of SCA remain unknown. There is consensus that ACh testing should be performed only when there is a strong clinical suspicion of spasm to reduce the risk of a false-positive result.^54^ Using new technologies for measuring the coronary flow reserve and microvascular resistance could help diagnosing coronary artery spasm,^3^ but this modality too has not yet been evaluated in SCA-survivors.

### Treatment

Managing coronary artery spasm involves lifestyle modifications, pharmacological treatment, and consideration of device implantation. Survivors should be counselled that smoking cessation is crucial, as smoking is associated with significantly worse survival outcomes.^1,63^

### Pharmacological treatment

Coronary artery spasm are typically managed with CCBs, either alone or in combination with different CCBs and/or long-acting nitrates.^10^ CCBs can suppress symptoms and VA-episodes,^30,53^ with non-dihydropyridine CCBs demonstrating superiority through lower incidence of ICD shocks in one study.^30^ Conversely, a reduction or cessation of medication may contribute to a rebound phenomenon, leading to high risk of recurrent events^21,22,30^ and suggestively a 10-fold increase in risk of cardiac death and nonfatal heart attack.^22^ Survivors are regularly treated with numerous vasodilators (mean between 1.5–2.6),^29,30,35,36^ but the effects of combination therapy or long-acting nitrates were not directly reported. Short-acting nitrates are known to provide symptom relief, possibly because the vasodilatory effects of nitrates are independent from endothelial integrity,^64^ making them effective despite dysfunctional endothelium that might be implicated in coronary artery spasm. Yet, the beneficial effect of long-lasting nitrates is debatable according to some studies.^10^ Treatment with β-blockers is contraindicated^30,53^ as inhibition of vasodilatory β-adrenergic receptors could exacerbate arterial spasm by transforming a sympathetic stimulus into an α-adrenergic vasoconstrictive response. Also, β-blockers might counteract the favourable effects of CCBs.^30^

Despite intensive medical treatment, a high incidence of recurrent VAs/SCA has been observed.^22,29,33^ This is supported by electrophysiological studies (EPS) by Komatsu *et al*. and Takagi *et al*. where 50% and 70% of the patients still had inducible VAs despite high-dose CCB therapy.^21,28^ Both studies proposed the so-called ‘*dual induction*’ tests (i.e. performing provocative testing alongside EPS) in survivors without structural heart disease to better understand the underlying pathophysiology.^21,28^ Dual induction strategies may identify the underlying mechanisms and guide treatments. A positive provocative test followed by a negative EPS may be suggestive of pure ischaemia from the vasospasm, implying the need of pharmacotherapy as the primary treatment approach. Conversely, if VAs were also induced during EPS, that would represent an indication for placing an implantable cardioverter-defibrillator (ICD).^28^ Notably, current guidelines do not include EPS as part of the diagnostic protocol for coronary artery spasm, possibly as EPS remain controversial in other conditions such as Brugada syndrome, where its diagnostic utility is debated.^53^ Repeating provocative testing to decide the effectiveness of optimal CCB dosages and of combined vasodilators has only been performed on small study populations^16,18,35,36^ and is not part of current guidelines. Evidence comparing CCB efficacy on long-term outcomes in patients with vasospastic angina (not on survivors) shows conflicting results and has important limitations, including retrospective design, lack of randomized trials, and potential confounding from selection bias and variable dosing.^10,65,66^

In summary, CCBs (including combination of different CCBs) appear to be the first choice of therapy, while β-blockers should be avoided. The beneficial effect of long-lasting nitrates remains uncertain. More clear recommendations regarding the choice of medicine or optimal dosages do not exist.

### When is ICD implantation needed?

Medical therapy alone may not be sufficient to prevent recurrent and potentially fatal VAs in SCA survivors with coronary artery spasm. In an Asian case series, the recurrence rate was 30% during 3.2 years follow-up.^38^ ICD treatment may offer additional protection, but clear indications for implantation remain undefined. The ESC guidelines on VAs and SCD suggest that ICD ‘should be considered’ in SCA- survivors with coronary artery spasm (a Class IIa recommendation),^53^ yet this recommendation provides limited practical guidance. The literature suggests, that ICD therapy in addition to medical treatment should be considered in SCA survivors with overlapping disorders due to an increased risk of recurrent VAs,^27,30,38,40,67,68^ or in case of poor medical compliance.^27^ Beyond these considerations, there is presently no validated method for predicting who will benefit most from ICD implantation. However, specific electrocardiographic patterns may help identify high-risk patients. Increased QT dispersion was observed in coronary artery spasm cases complicated by SCA or syncope, and may serve as both a diagnostic marker for VAs and as risk marker for mortality.^19^ Similarly, J waves on the electrocardiogram, particularly when spatially concordant with the location of coronary artery spasm, may help identify patients at increased risk of VAs.^67,69^

**Figure 1:**
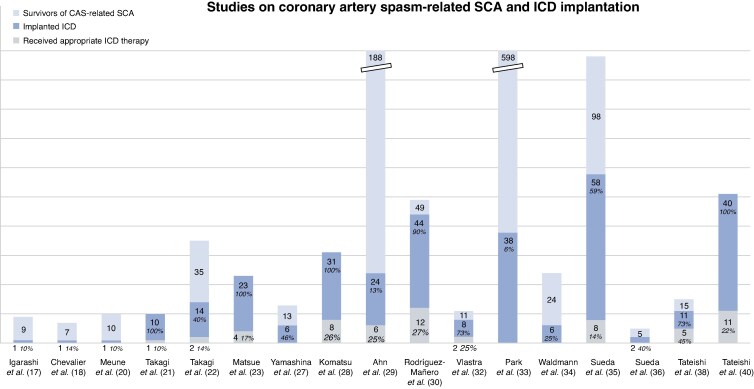
**ICD implantation and appropriate therapy delivery across studies.** In In four studies, data about ICD therapies were not available.^17,20,33,41^ In the study by Waldmann *et al*. only 24 out of 31 patients with coronary artery spasm-related SCA were alive at hospital discharge, which explains why seven patients are excluded from this chart.^34^ CAS, coronary artery spasm; ICD, implantable cardioverter defibrillator; SCA, sudden cardiac arrest.

Out of the 27 papers, 17 studies (63%) reported on ICD implantation treatment.^17,18,20–23,27–30,32–36,38,40^  *Figure 1* illustrates the distribution of survivors of coronary artery spasm-related SCA, who were implanted with an ICD and the number of reported appropriate ICD therapies. ICD implantation ranges from 6.3 to 100% across cohorts. Since most studies were retrospective observational in design, it is not possible to know exactly why clinicians decided for or against implantation.^33^ Patient preference,^29^ ICD availabilities as well as varying indications across countries and regions may also be relevant factors. Appropriate therapy included both anti-tachycardia pacing and/or defibrillation treatment for VAs that had not terminated spontaneously, and was mainly due to recurrent ventricular fibrillation.^21–23,28,29,32,35^ Therapy rates and follow-up duration varied considerably: The highest rate of appropriate therapy occurred in 45% of patients during a mean of 38 months of follow-up.^28,38^ Similar follow-up periods reported a therapy rate of 14–27%.^22,28^ The studies with the longest follow-up (of mean 90 months) found that 25% of patients had received appropriate therapy,^29,32^ while the study with the largest cohort of patients with implanted ICDs did not provide information regarding therapy, but solely reported no recurrent SCA-events in the patients.^33^ In one study, the average time from ICD insertion to appropriate therapy was reported to be about 12 months (range 50–600 days; median 292 days), but no significant differences in clinical characteristics were found between patients with or without recurrent events.^23^ These relatively high rates of new VAs/SCA-events in survivors contrast with recent reports showing that patients with ANOCA disease face a similar 15-year risk of major adverse cardiovascular events (myocardial infarction and mortality) compared to a matched general population cohort without known coronary artery disease.^70^ This disparity suggests a distinct, more severe phenotype in survivors that remains poorly defined.

The variance in reported number of appropriate ICD therapy could be due to the heterogeneity of pharmacological intervention and concomitant disorders. Inadequate anti-arrhythmic medication and suspected cocaine abuse could also be potential causes.^30,32^

Concerning inappropriate ICD therapy, two studies reported comparable rates of 9.1% and 10%.^30,40^ There was only one single mentioning of a death due to intractable ventricular fibrillation.^29^ Details about deaths in study subjects were rarely specified. A lower incidence of cardiac death in patients who received an ICD has been described, although not significantly, with an incidence rate of recurrent VAs to 32.4 per 1000 patient-years.^29^ Another study found a significantly lower recurrence of cardiac arrest and also acute myocardial infarction in SCA survivors who were implanted with an ICD, than in those without.^33^

In conclusion, pharmacological treatment alone may not be sufficient to prevent recurrent VAs, why ICD implantation may be warranted in most cases.

### Is coronary artery spasm causing SCA, or is SCA causing coronary artery spasm?

The pathophysiologic role of coronary artery spasm in SCA is incompletely understood.^5^ It is plausible that coronary vasospasm could act as a trigger or precipitating factor for electrical storms, given that these arrhythmic events can be provoked by acute myocardial ischaemia as well as external factors (such as hypothermia and fever, which also are known triggers in Early repolarization syndrome and Brugada syndrome respectively).^71^ This possibility is particularly relevant since primary electrical disorders appear to overlap with coronary artery spasm in patients with aborted SCD (in up to 58.8%).^21,26,28,38^ However, the European Heart Rhythm Association consensus statement on electrical storm and VA management does not explicitly address this potential mechanism.^71^ Coexisting Brugada syndrome in patients with both out-of-hospital cardiac arrest and coronary artery spasm was frequently diagnosed with an incidence rate between 7.4% and 51.6%.^21,26,28^ These patients have a higher frequency of recurrent ventricular fibrillation and a poorer prognosis compared to patients with coronary artery spasm alone.^28^ Early repolarization syndrome was present in 26–48.5% survivors with coronary artery spasm.^26,28,38^ Lee *et al*.^26^ found that nearly half their patient population (i.e. 46.2%) with recurrent SCD showed early repolarization with horizontal/descending ST segment, and identified this as the strongest predictor of recurrent SCD with an adjusted hazard ratio of 4.41 (95% confidence interval 1.26–15.40, *P* = 0.020). Tateishi *et al*.^38^ discovered that 40% of their resuscitated patients with a positive ACh test had other concomitant cardiac disorders. These disorders included Long QT syndrome, myocarditis, cardiac sarcoidosis, Brugada syndrome and lamin A/C-related cardiomyopathy. In their analysis of the long-term prognosis (mean follow-up was 3.2 ± 2.2 years), there was no statistically significant difference between patients with a positive ACh test with/without overlapping cardiac disorders, although the risk of recurrent SCA was found to be numerically higher in patients with coronary artery spasm.^38^

The prevalence of coronary artery spasm among survivors of unexplained SCA may be generally under-estimated since provocative testing is not conducted systematically.^20,34^ Only two studies performed provocative testing on all their patients.^21,28^ Both used ACh and found a high positive percentage, respectively 66% and 83.3%. Vlastra *et al*. studied a population with either acute coronary syndrome or out-of-hospital cardiac arrest, all suspected to be secondary to coronary artery spasm.^32^ In patients with evident coronary spasm, provocative testing was considered redundant. Therefore, just half of their population underwent ACh testing, yet all were found to be positive for coronary artery spasm. It is challenging to document direct evidence of coronary artery spasm causing SCA.^27,40^ It is known that endothelial injury is common in patients with aborted SCA.^72,73^ Vasospasm may also be a consequence of SCA, underlining the case for prospective studies.

### Gaps in evidence

**Figure 2:**
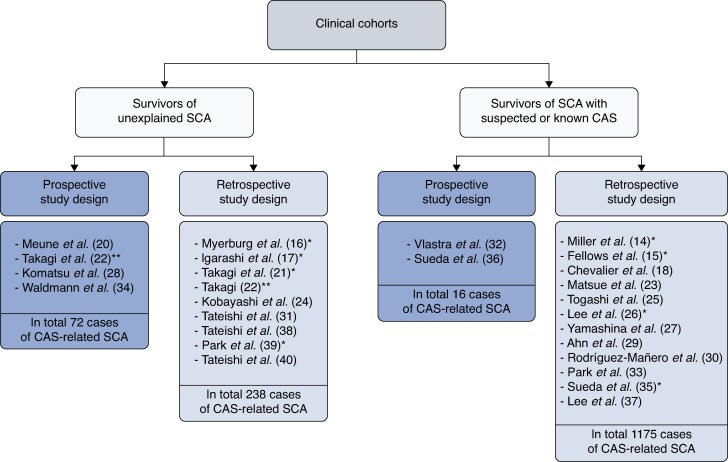
**Characteristics of the patients and study design of trials assessing survivors of SCA.** The reference to each publication is given in parentheses. *) Exact study design method was not specified but interpreted as being retrospectively. **) Patients in the Takagi *et al*. study^22^ were not included in the calculations above as it is not known how many were included retrospectively and how many recruited prospectively. The study by Parchure *et al*.^19^ is not listed as the study design could not be determined. CAS, coronary artery spasm; SCA, sudden cardiac arrest.

Studies on coronary artery spasm vary widely in their patient populations, designs, and methods. This heterogeneity makes it difficult to interpret current knowledge about prevalence, incidence, and mechanisms, or to derive clinically useful conclusions. The evidence base consists entirely of observational studies. In some studies, the enrolled patients did not initially have an identifiable cause of SCA, whereas in others, the population comprised pre-selected groups of patients with either suspected or known coronary artery spasm. Nonetheless, the number of patients with coronary artery spasm-related SCA is relatively small despite data collection over a long period of time, suggesting that coronary artery spasm is a relatively rare cause of SCA, the diagnosis may have been missed, or a combination of the two. ***Figure 2*** illustrates the characteristics of the patient populations and study designs. Only four studies of unselected populations had a prospective study design, including the study by Takagi *et al*.^22^ with data collected prospectively in only 1/3 of the study period.

Despite significant progress in recent years, our understanding of coronary artery spasm-induced SCA is incomplete, with many aspects of the problem requiring further research.^74^ Data on incidence and prevalence are limited across both Eastern and Western populations, but particularly the latter.^7^ Mechanisms explaining the male predominance of survivors are poorly understood. Gaps in evidence further persist regarding diagnostic strategies, sensitivity and specificity, the role of different provocative testing modalities and how and when provocative tests should be performed. Whether combining EPS with provocative testing to guide ICD implantation merits further research.

High-risk patients appear to be young male smokers without traditional coronary risk factors, with prior VAs/aborted SCD and/or overlapping electrical disorders. However, study heterogeneity, limits precise risk stratification. In accordance with the 2022 ESC guideline ‘*Algorithm for the evaluation of sudden cardiac arrest survivors’*, sodium channel blocker testing for Brugada syndrome is a Class I recommendation and precedes coronary artery spasm testing,^53^ No clear recommendations exist regarding simultaneous testing for both conditions or determining the dominant disorder when they coexist. More data are needed regarding patient risk stratification and indications for ICD insertion, as well as what represents optimal pharmacological therapy. The choice of ICD type remains unaddressed,^67^ though subcutaneous devices may be appropriate for survivors who do not require adjunctive pacing functions.^75^ Future investigations should include prospective studies and randomized trials to address these gaps in knowledge and improve management guidelines.^76^ Multicentre studies would have potential to increase the number of eligible patients to achieve better statistical power. Patient cohorts should be standardized regarding diagnostic workup and treatment protocols. Follow-up periods should be extended to maximize information. Future research should clarify the clinical significance of coronary artery spasm as a cause of SCA to better inform guidelines.

In conclusion, diagnosing coronary artery spasm as a cause of SCA is challenging due to an incomplete pathophysiological understanding and clinical guidelines are limited. Recurrent ventricular fibrillation in survivors with coronary artery spasm is a particular concern.

## Data Availability

No data were generated or analysed for or in support of this paper.
